# Comparable Hemodilution with Hypertonic Glucose in Patients with and without Type-2 Diabetes Mellitus during Hemodialysis

**DOI:** 10.3390/nu15030536

**Published:** 2023-01-19

**Authors:** Daniel Schneditz, Longin Niemczyk, Anna Wojtecka, Katarzyna Szamotulska, Stanisław Niemczyk

**Affiliations:** 1 Otto Loewi Research Center, Division of Physiology, Medical University of Graz, 8010 Graz, Austria; 2 Department of Nephrology, Dialysis and Internal Diseases, Medical University of Warsaw, 02-097 Warsaw, Poland; 3 Department of Internal Diseases, Nephrology and Dialysis, Military Institute of Medicine, 04-141 Warsaw, Poland; 4 Department of Epidemiology and Biostatistics, National Research Institute of Mother and Child, 01-211 Warsaw, Poland

**Keywords:** hemodialysis, glucose, osmotic effects, hemodilution, mathematical model, hemodynamics

## Abstract

(1) Background: It was examined whether glucose-induced changes in the relative blood volume are suitable to identify subjects with and without type-2 diabetes mellitus (T2D) during hemodialysis. (2) Methods: The relative blood volume was continuously recorded during hemodialysis and perturbed by the infusion of glucose comparable to the dose used for intravenous glucose tolerance tests. Indices of glucose metabolism were determined by the homeostatic model assessment (HOMA). Body composition was measured by a bioimpedance analysis. The magnitude and the time course of hemodilution were described by a modified gamma variate model and five model parameters. (3) Results: A total of 34 subjects were studied, 14 with and 20 without T2D. The magnitude of the hemodilution and the selected model parameters correlated with measures of anthropometry, body mass index, absolute and relative fat mass, volume excess, baseline insulin concentration, and HOMA indices such as insulin resistance and glucose disposition in a continuous analysis, but were not different in a dichotomous analysis of patients with and without T2D. (4) Conclusions: Even though the parameters of the hemodilution curve were correlated with measures of impaired glucose metabolism and body composition, the distinction between subjects with and without T2D was not possible using glucose-induced changes in the relative blood volume during hemodialysis.

## 1. Introduction

Undoubtedly, glucose is one of the prime energy substrates as it is easily delivered and distributed throughout the body without modification, enterally, parenterally, and in aqueous solutions. The water solubility of glucose, however, implies an osmotic activity and a deviation in the plasma glucose concentration from reference concentrations in chronic diseases or from baseline after parenteral infusion, which induces a perturbation in the osmotic equilibrium and the related body fluid and volume control [1]. Indeed, the physiology of normal glucose disposal can be seen as a process to minimize the osmotic perturbation throughout the body and to prevent wasteful losses of both glucose and water in the kidneys. The secretion of insulin, the insertion of glucose channels into otherwise glucose-tight cell membranes, and the disposal of glucose in insulin-dependent skeletal muscle and fat tissues constitute an essential part of that process. This part of glucose disposal is typically impaired with type-2 diabetes mellitus (T2D). The administration of glucose, either orally or as an intravenous glucose tolerance test, and the analysis of the time course of the resulting blood glucose concentrations is therefore used to screen for an impaired glucose metabolism in suspected individuals [2,3]. In clinical medicine, glucose is therefore used as a nutrient, a diagnostic tool, as well as an osmotic agent, to modify and adjust body water distribution and balance.

Both metabolic and osmotic effects of glucose are of special interest in chronic kidney disease and hemodialysis. The first, because of the prevalence and risk of impaired glucose metabolism in this group of patients [4,5,6]. The second, because of the volume balance and the transient osmotic effect [7].

The metabolic effects of parenteral, hypertonic glucose infused into subjects with and without T2D during hemodialysis have been analyzed in a preceding study [8]. The differences in glucose kinetics during a one-hour observation phase were only small because the early phase of glucose distribution is not controlled by insulin-dependent disposal in poorly perfused skeletal muscle and fat tissues. This phase is controlled by an extracorporeal removal by high-efficiency dialysis as well as by the same regional blood flow distribution physiologically shared by both subjects with and without T2D.

The osmotic and hypervolemic effects of hypertonic glucose infused during hemodialysis have also been studied before [9]. In a small group of chronic hemodialysis patients without T2D, the infusions produced a significant hemodilution, an increase in stroke volume, a decrease in peripheral resistance, and no change in blood pressure or heart rate. The hemodilution resolved within about 40 min after the infusion and corresponded with arterial glucose concentrations.

This leads to the question whether the time course of osmosis-induced hemodilution, which is easily measured during dialysis using available online technology, is different in subjects with impaired metabolic and cardiovascular control, and whether osmotic hemodilution is suitable to identify the differences in the metabolic parameters.

It therefore was the aim of this study to analyze the volume kinetics of a bolus of concentrated glucose that is delivered directly into the extracorporeal blood stream during routine hemodialysis to examine the hypervolemic effects and possible differences between subjects with and without T2D.

## 2. Materials and Methods

This is the continuation of an explorative study on glucose and insulin kinetics performed in the same group of hemodialysis patients, with and without T2D, reported previously [8,10]. More information about material and methods is found in these publications. The focus of this study is on osmotic effects of hypertonic glucose on blood volume.

### 2.1. Subjects

The study was performed on hemodialysis patients from the dialysis program of the Military Institute of Medicine in Warsaw, as described previously [8,10]. Patients older than 18 years, on hemodialysis for more than three months, who provided written informed consent, but without clinical signs of infection, symptomatic anaemia, wasting disease, hormonal therapy, or diabetogenic drug prescription were eligible to participate in the study. Subjects without T2D were identified as the control group, henceforth termed “Without”, subjects with T2D were identified as the study group, henceforth termed “With”.

Participants were studied during their regular hemodialysis treatment. Participants were asked to refrain from eating and drinking more than 3 h prior to starting hemodialysis and to assume a supine body position for the duration of the test. Individual medication was maintained as prescribed, with the exception of insulin, which was withheld for 8 h before starting the study in subjects with T2D to avoid interference with glucose disposition.

### 2.2. Protocol

Hemodialysis was delivered using standard dialysis equipment and procedures, as described previously [8,10]. All subjects received the same treatment and there was no randomization. Approximately 30 min after having started hemodialysis, a 40% aqueous solution of glucose was injected into the venous drip chamber at a constant rate of 1 mL/s (Angiomat 6000, Liebel-Flarsheim, Cincinnati, OH, USA) to deliver a glucose load of 0.5 g per kg target body mass (so-called dry weight). This dose corresponds to the glucose load of an intravenous glucose tolerance test, typically ranging between 0.3 and 0.5 g/kg [11,12,13]. As subjects are volume expanded at the beginning of dialysis, we chose the higher dose of 0.5 g/kg. The beginning of the infusion was marked as time *t* = 0.

Relative blood volume (*RBV*, in %) was continuously recorded by the ultrasonic blood volume monitor (BVM, Fresenius Medical Care, Schweinfurt, Germany) with a sampling period of 10 s [14].

Baseline glucose (*c*_g_, in mmol/L) and insulin concentrations (*c*_i_, in mU/L) measured at the beginning of dialysis (*t* = −30 min), as described elsewhere [8,10], were used for the calculation of homeostatic model assessment (HOMA) indices to estimate insulin resistance (*R*_HOMA_ = *c*_g_ × *c*_i_/22.5), ß-cell function (*ß*_HOMA_ = 20 × *c*_i_/(*c*_g_ − 3.5)), insulin sensitivity (*S*_HOMA_ = 100/*R*_HOMA_), and disposition (*D*_HOMA_ = *S*/*ß*_HOMA_).

The body composition monitor (BCM, Fresenius Medical Care, Schweinfurt, Germany) was used to measure extracellular water, intracellular water, total body water, fat mass, fat mass percentage, lean tissue mass percentage, and volume excess before dialysis.

Arterial pressures and heart rates were manually measured by cuff technique.

### 2.3. Glucose Dilution

The volume effect of hypertonic glucose infused at *t* = 0 was derived from relative blood volume data continuously recorded throughout the treatment. The original relative blood volume curve recorded from the beginning of the treatment (at *t* = −30, and where *RBV* = 100% by definition) was resampled with a sampling period of 1 min, and was rescaled to the time of infusion (*t* = 0) by dividing recorded *RBV*_t_ data by relative blood volume at time of infusion (*RBV*_0_):

(1) $$V_{rel,t}=100\frac{RBV_{t}}{RBV_{0}}$$

So that *V*_rel,t_ was 100% at the beginning (*t* = 0) of each dilution curve.

The resulting dilution curve *V*_rel,t_ was then analyzed and compared in individual treatments.

The shape and magnitude of the dilution curve were analyzed using a combination of the gamma variate function [15] and a linear function:

(2) $$V_{rel,t}=A{\left(t-\vartheta \right)}^{\alpha }e^{\left(-\left(t-\delta \right)/\beta \right)}+d-kt$$

where *t* refers to time, *δ* refers to delay from *t* = 0 to the appearance of indicator, *A*, *α*, and *ß* refer to the model parameters of the gamma variate function, and where *d* and *k* refer to the intercept and the slope of the linear function. As the relative blood volume curve is scaled to *RBV*_0_ = 100% at *t* = 0, the parameter *d* = 100 by definition.

### 2.4. Data Analysis

The five parameters (*δ*, *A*, *α*, *ß*, and *k*) of the dilution function (2) were identified by non-linear least square analysis, and by fitting the dilution function to experimental data using the Levenberg–Marquardt algorithm provided by Kaleidagraph vs. 5.0 software (Synergy Software, Reading, PA, USA). The quality of the model fit was assessed by Pearson correlation coefficient (*r*). Parameters were identified for the period from *t* = −5 min to *t* = 60 min after eliminating implausible records (*V*_dil,t_ < 99.9) from the data set for the very short time period 0 < *t* < 3 min immediately following the infusion of glucose.

### 2.5. Statistics

Data were analyzed by Microsoft Excel 16.16.27 and are presented as average ± standard deviation (SD) or as median and 25% and 75% quartiles, depending on type of data distribution examined by analysis of skewness and kurtosis. The correlation between variables was examined by Pearson correlation coefficients (*r)* after log-transformation of non-normally distributed variables and Fisher’s *r* to *z* transformation for estimation of probabilities. Hypothesis testing was conducted using StatView 4.5 software (Abacus Concepts Inc., Berkeley, CA, USA). Differences between subjects with and without T2D were examined by unpaired t-test or Mann–Whitney U-test, depending on data distribution. Differences in serial data were examined by ANOVA for repeated measurements, and Dunnett’s post-hoc test to account for multiple comparisons. A probability *p* < 0.05 was considered significant to reject the null-hypothesis (*H*_0_).

## 3. Results

Data were collected in 35 subjects, but the complete relative blood volume measurements were only available in 14 subjects with, and in 20 subjects without T2D, so that 34 datasets entered the final analysis (Table 1). Twelve of the thirty-four subjects were female, corresponding to 35.3% of the study population. Study groups were comparable with regard to the female to male distribution and other patient characteristics. with the exception of higher age (70.6 ± 7.9 vs. 55.0 ± 13.4 y, *p* < 0.001), higher baseline glucose (7.2 (5.3,11.1) vs. 5.4 (5.0,5.9) mmol/L, *p* < 0.05), larger volume excess (4.6 ± 3.3 vs. 1.9 ± 2.3 L, *p* < 0.01), lower relative lean tissue mass (41.3 ± 8.8, vs. 52.6 ± 12.0%, *p* < 0.01), and lower ß-cell function (61.0 (40.2,97.8) vs. 148.1 (92.0,189.4), *p* < 0.05) in subjects with T2D.

**Figure 1:**
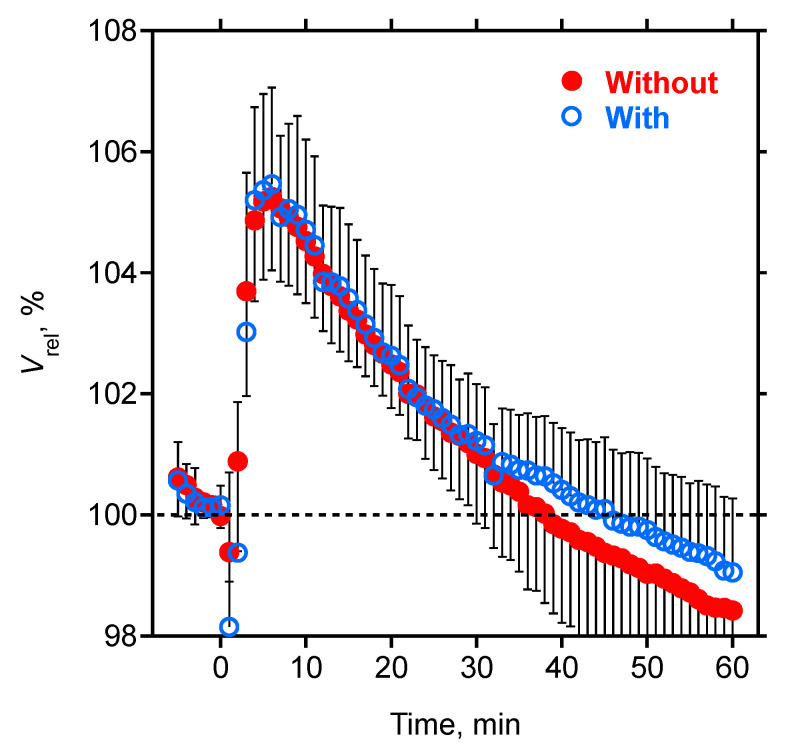
Relative hemodilution. Relative blood volume (*V*_rel_ ± SD) in subjects without (closed symbols, *n* = 20) and with (open symbols, *n* = 14) T2D, relative to *V*_rel,0_ = 100% at the time of glucose injection (*t* = 0). T2D, type-2 diabetes mellitus.

**Figure 2:**
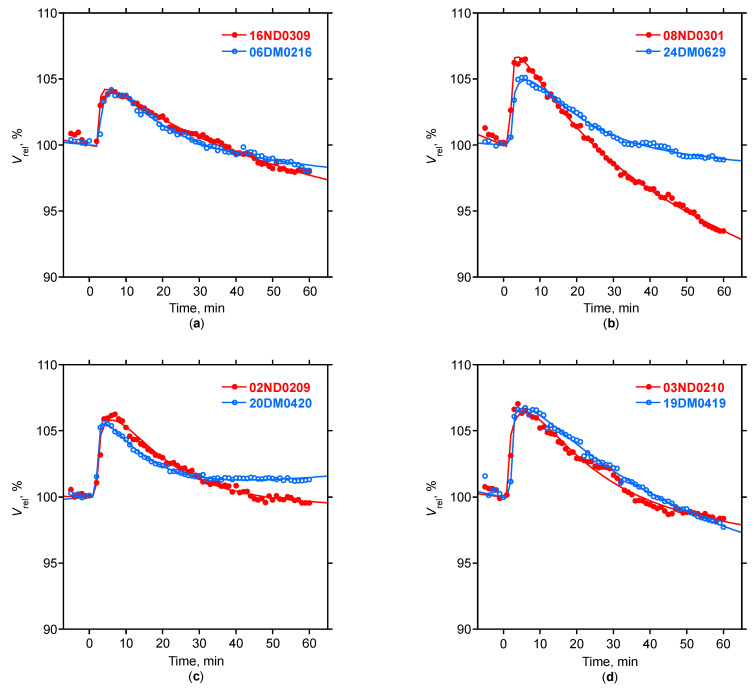
Relative blood volume modeling. Relative blood volume data (symbols) and corresponding non-linear model fits (full lines) of eight dilutions obtained in four subjects with (open symbols) and without (full symbols) T2D. The legends refer to study numbers. Pearson correlation coefficients (*r*) and model parameters (*δ*, *A*, *α*, *ß*, and *k*) are provided for each study: (**a**) #16 (Without): *r* = 0.99, *δ* = 2.99, *A* = 4.56, *α* = 0.08, *ß* = 25.61, and *k* = 0.05; #06 (With): *r* = 0.99, *δ* = 2.99, *A* = 3.9, *α* = 0.31, *ß* = 11.19, and *k* = 0.03; (**b**) #08 (Without): *r* = 1, *δ* = 2, *A* = 7.35, *α* = 0.13, *ß* = 16.56, and *k* = 0.11; #24 (With): *r* = 1, *δ* = 1.99, *A* = 4.25, *α* = 0.37, *ß* = 11.72, and *k* = 0.02; (**c**) #02 (Without): *r* = 0.99, *δ* = 1.99, *A* = 4.98, *α* = 0.36, *ß* = 12.04, and *k* = 0.01; #20 (With): *r* = 1, *δ* = 2, *A* = 5.87, *α* = 0.17, *ß* = 11.02, and *k* = −0.02; (**d**) #03 (Without): *r* = 0.99, *δ* = 1, *A* = 5.12, *α* = 0.39, *ß* = 13.29, and *k* = 0.04; and #19 (With): *r* = 1, *δ* = 2, *A* = 6.58, *α* = 0.17, *ß* = 24.92, and *k* = 0.06.

The infusion of hypertonic glucose was completed after 104.6 ± 18.9 s in subjects with, and 98.2 ± 24.1 s in subjects without T2D, and led to a pronounced and significant increase in the relative blood volume in all subjects (Figure 1). The relative blood volume peaked about 6 min after the infusion and was comparable in subjects with and without T2D (Table 2). The relative blood volume curve then slowly declined, and after 40 to 50 min, the resumed values were no longer different from those at *t* = 0. The relative blood volume curve then further declined below baseline within 60 min of observation. Individual traces showed considerable variability in the shape and the magnitude of the dilution curve (Figure 2), but the average *V*_rel,t_ profile was not different in subjects with or without T2D (Table 2).

All dilution curves were adequately described by the non-linear model described in (2), as documented by the correlation coefficients *r* > 0.98 (Table 2). The model parameters were not different between the subjects without or with T2D (Table 2).

Peak dilution *V*_rel,6_ and the parameters of the *V*_rel,t_ model correlated with measures of anthropometry, body composition, and glucose metabolism (Table 3). *V*_rel,6_ showed positive correlations with measures of obesity such as body mass index, fat mass, and relative fat mass (*p* < 0.001), and showed negative correlations with relative lean tissue mass and volume excess (*p* < 0.001). Furthermore, *V*_rel,6_ positively correlated with the baseline insulin concentration and insulin resistance, and negatively correlated with the insulin sensitivity and glucose disposition (*p* < 0.001). Similar correlations were observed between model parameter *A* and the aforementioned measures of anthropometry, body composition, and glucose metabolism (Table 3). The single strongest positive correlation was observed between *A* and peak dilution *V*_rel,6_ (*r* = 0.88, *p* < 0.001). which also explains their similarity in the observed correlations. Model parameter *α* was negatively correlated with model parameters *δ* and *ß* (*p* < 0.001). Model parameter *ß* was weakly correlated with the relative fat mass and relative lean tissue mass (*p* < 0.05). Model parameter *k* was weakly correlated with the baseline insulin concentration, insulin resistance, insulin sensitivity, and glucose disposition (*p* < 0.05).

**Figure 3:**
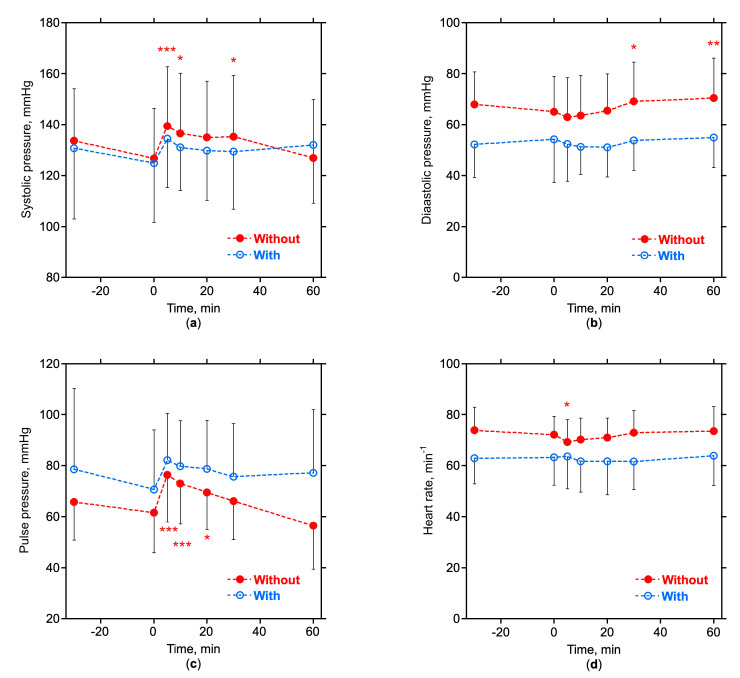
Arterial pressures and heart rates. Average ± SD data in subjects without (closed symbols) and with T2D (open symbols). Glucose is injected at time *t* = 0. Broken lines indicate interpolations between measuring points; (**a**) systolic pressures; (**b**) diastolic pressures; (**c**) pulse pressures; (**d**) and heart rates; *, *p* < 0.05; **, *p* < 0.01, and ***, *p* < 0.001 different from value at *t* = 0 (ANOVA for repeated measurements and Dunnett’s post-hoc test).

All studies were completed without symptoms. Systolic pressures were comparable in both groups whereas diastolic pressures (*p* < 0.05) and heart rates (*p* < 0.01) were significantly lower in subjects with T2D at the time *t* = 0 of glucose infusion (Table 2) and for most of the test duration (Figure 3). Infusion of glucose led to a transient increase in the systolic blood pressure of the subjects without T2D. This was accompanied by a transient decrease in heart rate. The transient decrease in diastolic pressure following the infusion of glucose was not significant, and the diastolic pressure increased during the last half hour of the observation phase in subjects without T2D (Figure 3).

## 4. Discussion

This study examined the magnitude and the time course of hypertonic glucose-induced hemodilution in subjects with and without T2D during hemodialysis. While there were no differences in hemodilution between subjects with and without T2D, the peak dilution *V*_rel,6_ and the parameter *A* of the hemodilution model were moderately correlated with measures of anthropometry, body composition, and glucose metabolism.

About one third of the study population (35.3%) was female, and a low, albeit comparable, female representation was also found in both study groups (Table 1). The lower percentage of female patients is not untypical for the general hemodialysis population, where the prevalence of kidney replacement therapy is higher in men, even though the prevalence of kidney disease is higher in women [16]. This paradoxical discrepancy has attracted much interest and speculation. It is also surprising that insulin resistance determined by the homeostatic model assessment at baseline was not different between the study groups. One explanation is a higher insulin resistance level in dialysis patients without diabetes [17,18]. Moreover, as discussed previously, a true fasting state was probably not really achieved in many subjects, leading to elevated glucose and insulin concentrations at baseline and thereby to inflated insulin resistance by the homeostatic model assessment [8].

Glucose is not a passive solute. Glucose is utilized to deliver electrolyte-free water in cases of dehydration using (close to) isotonic glucose infusions, or to stimulate a transient osmosis-induced expansion of the extracellular volume in cases of hypovolemic hypotension using hypertonic glucose infusions. Most importantly, as glucose is eventually disposed of and metabolized, the delivery of glucose does not produce a long-term solute perturbation, such as observed with the administration of normal or concentrated saline. This aspect is of interest in hemodialysis, as it avoids the accumulation of sodium.

The relative blood volume increased by about 5% following the infusion of 100 mL of hypertonic glucose solution (40%), which was much larger than the expected 2% (0.02 = 100/5000) anticipated for an absolute blood volume in the range of 5 L and with a neutral indicator, such as isotonic saline or dialysate [19]. The large volume effect has been observed before and has been explained by osmotic effects [7,9].

The peak dilution of about 5% was observed 6 min after starting the glucose infusion. At that time. the glucose bolus will have equilibrated throughout the blood and within the central parts of the extravascular compartment because glucose is not confined to the vascular space. An assumed osmotic fluid shift within the vascular space, from red blood cells to plasma, lowers the hematocrit and increases the plasmatocrit, but has no net effect on overall blood volume. The relative blood volume sensor used in this study measures the total protein concentration in whole blood and is insensitive to fluid shifts between plasma and red blood cells [20].

The dilution in excess of 2% therefore has to originate from the extravascular space. The exact mechanism for osmotic refilling from the extravascular space remains unclear. It is generally believed that osmotic refilling is a consequence of fluid shifts from intra- to extracellular spaces [7,21,22,23,24], and that vascular refilling increases secondary to the increase in the interstitial volume. An osmosis-induced increase in interstitial volume, however, is accompanied by an identical decrease in intracellular volume, so that the overall extravascular volume remains unchanged as well. The rapidity and magnitude of osmotic refilling therefore suggest that refilling occurs by osmotic forces across the microvasculature, comparable to much smaller colloid-osmotic gradients, first within the central, highly perfused fraction of intra- and extravascular spaces before glucose equilibrates throughout the extracellular compartment [22].

Glucose is not a passive solute with regard to humoral effects. Glucose stimulates the secretion of insulin in the pancreas, with specific metabolic and hemodynamic consequences. Apart from expected differences in the secretion and glucose utilization in subjects with and without T2D, the glucose bolus caused only small differences in the overall glucose and insulin balance during hemodialysis, as analyzed in a preceding study [10]. Insulin is known to induce a vasodilatation in the large insulin-dependent skeletal muscle tissue compartment [25]. Acute hyperglycemia, however, impairs muscle microvascular blood flow and vasodilatation, potentially limiting glucose disposal into the skeletal muscle [26]. Without vasodilatation the resting muscle remains poorly perfused, skeletal muscle glucose uptake is delayed, and the systemic glucose concentration remains elevated. A drop in arterial blood pressures was not observed in this study, neither in subjects with nor in subjects without T2D, most likely because of the large volume effect of hypertonic glucose and also probably because of the vasoconstrictive effect of acute hyperglycemia [26]. Quite on the contrary, the arterial pressures increased and the heart rates decreased in subjects without T2D, indicating a functional baroreflex response. The hypervolemic effect of glucose is also expected to increase venous return, stroke volume, and cardiac output. An increase in cardiac output of about 20% following the infusion of hypertonic glucose infusion has indeed been measured in hemodialysis patients without T2D in a preceding study [9]. The increase in pulse pressure in this study is also indicative of increased stroke volume (Figure 3). A more detailed analysis of the hemodynamic effects of hypertonic glucose is presented in the companion publications of the same journal [27,28].

Glucose is not a passive solute with regard to neuroendocrine effects. The rapid infusion of concentrated glucose increases the osmotic pressure, especially in highly perfused regions of the body such as the brain, and is expected to stimulate the osmoreceptors and the secretion of vasopressin. The resulting increase in arterial pressures confirms the observations by Shimizu et al., where the rapid infusion of a small volume (20 mL) of 50% *w/v* glucose solution stimulated the osmotic release of vasopressin and thereby increased arterial pressures without any significant volume effect [29].

The similarity of the relative blood volume curves in subjects with and without T2D suggests that the osmotic effects of glucose and the hemodynamic effects of insulin are of secondary importance with parenteral glucose administration during hemodialysis. This also confirms the observation of comparable glucose kinetics in subjects with and without T2D, analyzed in a companion paper [8]. In this previous study, the similarity of glucose kinetics was explained by comparable flow-controlled transport throughout the vascular system during the early phase of glucose dispersion, as well as comparable extracorporeal glucose clearance, which removed about 30% of the glucose bolus within the whole observation phase in both subjects with and without T2D. It follows that impaired glucose disposal is masked by extracorporeal glucose removal, which also serves a type of safety overflow.

Relative blood volume curves were analyzed by a gamma variate model (2) modified for a constant rate *k* in the relative blood volume change, to account for effects caused by ongoing ultrafiltration and vascular refilling. This modification allows *V*_rel,t_ to drop below baseline during the observation phase (Figure 2). Model parameters were easily identified by a non-linear least square fitting. Indeed, highly acceptable fits were obtained in all studies, as the correlation coefficient was better than 0.98 for most dilution curves. Parameter *A* in this model is a scaling factor and is mathematically related (but not identical) to the amplitude of the dilution curve. The strong correlation between *A* and *V*_rel,6_ is therefore expected (*r* = 0.88, Table 3). Both *V*_rel,6_ and *A* were moderately correlated with body mass index, fat mass, and relative fat mass. This is also plausible because the (aqueous) distribution volume for glucose is smaller in subjects with larger lipid content (as concentrations are inversely related to the aqueous distribution volume). Consequently, *V*_rel,6_ and *A* were negatively correlated with the relative lean tissue mass and volume excess, as both variables increased the (aqueous) distribution volume. *V*_rel,6_ and *A* were both moderately correlated with the baseline insulin concentration, insulin resistance (*R*_HOMA_), insulin sensitivity (*S*_HOMA_), and glucose disposition (*D*_HOMA_) derived from the homeostatic model assessment (Table 3). Parameter *α* was moderately and inversely correlated with the delay *δ* of the dilution curve. Parameter *ß* was weakly correlated with the relative lean tissue mass and inversely correlated with the relative fat mass. A physical representation of the gamma variate model has been presented for the dispersion of the indicator in tube flow [30], but a mechanistic interpretation of model parameters *α* and *ß* remains to be developed for the volume kinetics studied in the setting of this study. The model parameter *k* is not part of the gamma variate model and describes the continuous ultrafiltration-induced decline of relative blood volume. The parameter *k* was weakly correlated with insulin resistance and inversely correlated with glucose disposition (Table 3). Impaired glucose metabolism was therefore correlated with a faster decline in the relative blood volume curve during hemodialysis and ultrafiltration. This is opposite to the relative blood volume effect of delayed glucose disposition expected in subjects with T2D, thereby opposing and masking the volume effect of hyperglycemia and impaired glucose tolerance in subjects with T2D. This is another explanation for the lack of differences seen in hemodilution curves *V*_rel,t_ between subjects with and without T2D (Figure 1, Table 2), in spite of significant correlations of the selected model parameters with measures of body composition and impaired glucose metabolism (Table 3).

It is a limitation of the study that the reproducibility of the hemodilution test is unknown. Each subject was only studied once, and it is not clear which variability in hemodilution can be expected in the same subject under identical test conditions. This is especially important as the ultrafiltration requirements are known to affect relative blood volume and depend on inter-dialytic weight gain and vary throughout the week [31]. The association of volume excess with some of the hemodilution parameters (Table 3) indicates that the volume status of patients should have been controlled when investigating the glucose-insulin system with a relative blood volume method.

## 5. Conclusions

In conclusion, there were only small differences in the hemodilution directly determined from the relative blood volume changes recorded during an intra-dialytic glucose tolerance test performed on subjects with and without T2D. A more detailed analysis using a modified gamma variate hemodilution model revealed significant correlations between selected model parameters and measures of body composition and glucose metabolism. The clear identification of subjects with T2D and insulin resistance during everyday hemodialysis using available non-invasive relative blood volume techniques remains difficult. The intravenous administration of glucose during hemodialysis is also safe to use in diabetic hemodialysis patients.

## Figures and Tables

**Table 1:** Anthropometric and treatment data in subjects with and without T2D.

Variable (Unit)	With (*n* = 14)	Without (*n* = 20)	*p*
Female (number, percentage)	6 (43%)	6 (30%)	0.49
Age (years)	70.6 ± 7.9	55.1 ± 13.4	<0.001
Height (cm)	168.4 ± 9.6	169.3 ± 8.5	0.79
Dry body mass (kg)	83.7 ± 15.1	78.6 ± 19.2	0.41
BMI (kg.m^−2^)	29.5 ± 4.3	27.3 ± 5.7	0.24
Total body water (L)	39.7 ± 7.7	39.7 ± 8.3	0.97
Extracellular volume (L)	20.9 ± 4.3	18.9 ± 3.7	0.16
Intracellular volume (L)	18.8 ± 3.8	20.9 ± 4.9	0.19
Fat mass (kg)	34.2 ± 9.9	27.8 ± 12.7	0.12
Relative fat mass (%)	39.0 ± 9.2	32.8 ± 10.1	0.08
Relative lean tissue mass (%)	41.3 ± 8.8	52.6 ± 12.0	<0.01
Volume excess (L)	4.6 ± 3.3	1.9 ± 2.3	<0.01
Ultrafiltration volume (L)	2.5 ± 1.0	2.4 ± 0.9	0.85
Ultrafiltration rate (L/h)	0.3 ± 0.0	0.3 ± 0.0	0.11
Hematocrit (%)	34.6 ± 4.4	35.1 ± 2.7	0.71
Infusion volume (mL)	104.6 ± 18.9	98.2 ± 24.1	0.41
Baseline glucose (mmol/L)	7.2 (5.3,11.1)	5.4 (5.0,5.9)	<0.05
Baseline insulin (pmol/L)	72.3 (49.4,90.2)	83.9 (47.1,124.0)	0.36
Insulin resistance	3.6 (2.9,4.5)	3.2 (1.9,5.6)	0.62
ß-cell function	61.0 (40.2,97.8)	148.1 (92.0,189.4)	<0.05
Insulin sensitivity	27.8 (22.5,35.0)	31.2 (17.8,51.4)	0.62
Glucose disposition	0.4 (0.2,1.2)	0.2 (0.1,0.6)	0.21

Abbreviations: T2D, type-2 diabetes mellitus; *n*, number of subjects; *p*, probability to reject the null-hypothesis for differences between study groups (unpaired t-test, Mann–Whitney U-test).

**Table 2:** Hemodynamic variables and model parameters in subjects with and without T2D.

Variable (Unit)	With (*n* = 14)	Without (*n* = 20)	*p*
*V*_rel_ at *t* = 0, (%)	100.1 (100.0,100.3)	100.0 (99.9,100.1)	0.11
*V*_rel_ at *t* = 6, (%)	105.5 ± 1.6 ***	105.3 ± 1.2 ***	0.67
*V*_rel_ at *t* = 10, (%)	104.7 ± 1.5 ***	104.5 ± 1.0 ***	0.67
*V*_rel_ at *t* = 20, (%)	102.6 ± 1.2 ***	102.5 ± 0.7 ***	0.67
*V*_rel_ at *t* = 30, (%)	101.2 ± 0.9 *	101.0 ± 1.1*	0.57
*V*_rel_ at *t* = 40, (%)	100.4 ± 1.0	99.8 ± 1.6	0.19
*V*_rel_ at *t* = 50, (%)	99.7 ± 1.2	99.0 ± 2.0	0.22
*V*_rel_ at *t* = 60, (%)	99.1 ± 1.2 *	98.4 ± 2.4 ***	0.37
Model: Pearson *r*	0.99 (0.98,1.00)	0.99 (0.99,1.00)	0.96
Model: Delay, *δ* (min)	2.56 ± 0.65	2.33 ± 0.97	0.45
Model: Parameter *A*	5.47 ± 1.60	5.10 ± 1.40	0.47
Model: Parameter *α*	0.15 (0.11,0.28)	0.19 (0.11,0.29)	0.55
Model: Parameter *ß*	14.96 (11.46,22.62)	16.42 (12.04,25.85)	0.58
Model: Parameter *k*	0.03 ± 0.03	0.04 ± 0.04	0.40
Systolic pressure at *t* = 0, (mmHg)	124.9 ± 23.2	126.7 ± 19.8	0.81
Diastolic pressure at *t* = 0, (mmHg)	54.2 ± 16.9	65.1 ± 13.9	<0.05
Pulse pressure at *t* = 0, (mmHg)	70.6 ± 23.4	61.6 ± 15.8	0.22
Heart rate at *t* = 0, (min^−1^)	63.2 ± 10.8	71.6 ± 6.9	<0.01

Abbreviations: T2D, type-2 diabetes mellitus; *n*, number of subjects; *t*, time; *p*, probability to reject the null-hypothesis for differences between study groups (unpaired t-test, Mann–Whitney U-test); *δ*, *A*, *α*, *ß*, and *k*, model parameters; *, *p* < 0.05, and ***, *p* < 0.001, significantly different from *V*_rel_ at *t* = 0 (ANOVA for repeated measurements and Dunnett’s post-hoc test).

**Table 3:** Correlation coefficients of model parameters.

Variable	*V*_rel,6_	*δ*	*A*	*α*	*ß*	*k*
Age	−0.12	0.08	0.01	−0.09	0.05	−0.21
Height	−0.29	0.24	−0.36 *	0.07	0.04	−0.10
Dry body mass	0.40 *	0.29	0.20	0.13	−0.15	0.07
Body mass index	0.62 ***	0.21	0.44 **	0.10	−0.19	0.14
Fat mass	0.65 ***	0.21	0.47 **	0.16	−0.33	0.07
Relative fat mass	0.73 ***	0.16	0.63 ***	0.06	−0.40 *	0.03
Relative lean tissue mass	−0.58 ***	−0.14	−0.50 **	−0.11	0.37 *	0.07
Volume excess	−0.63 ***	0.03	−0.54 ***	0.11	0.15	−0.29
Ultrafiltration volume	−0.02	0.29	0.02	−0.09	0.26	0.33
Ultrafiltration rate	−0.11	0.28	−0.21	−0.19	0.19	−0.06
Baseline glucose	0.15	0.05	0.12	0.23	−0.29	0.07
Baseline insulin	0.58 ***	0.24	0.57 ***	−0.16	−0.11	0.37 *
Insulin resistance	0.55 ***	0.23	0.53 ***	−0.04	−0.21	0.35 *
ß-cell function	0.31	0.12	0.33	−0.29	0.12	0.23
Insulin sensitivity	−0.55 ***	−0.23	−0.53 ***	0.04	0.21	−0.35 *
Glucose disposition	−0.53 ***	−0.22	−0.54 ***	0.20	0.06	−0.36 *
*V*_rel,6_	1.00	−0.03	0.88 ***	0.05	−0.31	0.13
*δ*	−0.03	1.00	0.16	−0.68 ***	0.32	0.12
*A*	0.88 ***	0.16	1.00	−0.25	−0.20	0.27
*α*	0.05	−0.68 ***	−0.25	1.00	−0.57 ***	−0.16
*ß*	−0.31	0.32	−0.20	−0.57 ***	1.00	0.27

Abbreviations: *V*_rel,6_, peak relative blood volume at *t* = 6 min; *δ*, *A*, *α*, *ß*, and *k*, model parameters; *, *p* < 0.05; **, *p* < 0.01; ***, *p* < 0.001.

## Data Availability

Data are available on request from the corresponding author. The data are not publicly available due to the Polish General Data Protection Regulations.
